# Supplementary material for: The Systems Biology Research Tool: evolvable open-source software
Source: BMC Syst Biol. 2008 Jun 29;2:55. doi: 10.1186/1752-0509-2-55 (PMC2446383; doi:10.1186/1752-0509-2-55)
Supplement: Additional file 1 — SBRT Archive. An archive of the current version of the Systems Biology Research Tool. [file 1752-0509-2-55-S1.zip › sbrt-1.4.0/doc/developers_guide/api/sbrt/kernel/util/PercentProgressMonitor.html]

PercentProgressMonitor


|  |  |  |
| --- | --- | --- |
| **Overview** \| **Package** \| **Class** \| **Use** \| **Tree** \| **Deprecated** \| **Index** \| **Help** | |  |
| **PREV CLASS**   **NEXT CLASS** | **FRAMES**    **NO FRAMES**     **All Classes** |  |
| SUMMARY: NESTED \| FIELD \| CONSTR \| METHOD | DETAIL: FIELD \| CONSTR \| METHOD |  |


---


## sbrt.kernel.util Class PercentProgressMonitor

```
java.lang.Object
  sbrt.kernel.util.AbstractProgressMonitor
      sbrt.kernel.util.PercentProgressMonitor
```

**All Implemented Interfaces:**: MutableProgressMonitor, ProgressMonitor

---

``` public class PercentProgressMonitor extends AbstractProgressMonitor ```

This class is used to monitor a process whose progress can be measured as
a percentage.

**Author:**
:   This class was written and documented by
    Jeremiah Wright while in the Wagner lab.

---

**Constructor Summary**

`PercentProgressMonitor()`
  
          Constructs a new percentage-based progress monitor.

`PercentProgressMonitor(int total)`
  
          Constructs a new percentage-based progress monitor.


| **Method Summary** | |
| --- | --- |
| `ProgressMonitor` | `asUnmodifiable()`             Returns this progress monitor as an immutable object. |
| `void` | `complete()`             Sets the internal counter equal to the provided total. |
| `java.lang.Double` | `getProgress()`             Returns a double precision number indicating the completed percentage of the process being monitored. |
| `void` | `increment()`             Causes this progress monitor to increment an internal counter. |
| `void` | `resetCount()`             Causes this progress monitor to reset it's internal counter to 0. |
| `void` | `setTotal(int total)`             Sets the total number of times `increment()` can be called. |

**Methods inherited from class sbrt.kernel.util.AbstractProgressMonitor**

`getCount, getDuration, start, started, stop, stopped`

**Methods inherited from class java.lang.Object**

`clone, equals, finalize, getClass, hashCode, notify, notifyAll, toString, wait, wait, wait`

**Constructor Detail**

### PercentProgressMonitor

```
public PercentProgressMonitor()
```

:   Constructs a new percentage-based progress monitor.

---


### PercentProgressMonitor

```
public PercentProgressMonitor(int total)
```

:   Constructs a new percentage-based progress monitor.

    **Parameters:**: `total` - the maximum number of calls allowed for `increment()`. **See Also:**: `setTotal(int)`


**Method Detail**

### setTotal

```
public void setTotal(int total)
```

:   Sets the total number of times `increment()` can be called. This
    is the value for which computed percentages are relative to.

    :   **Parameters:**: `total` - the total number of times `increment()` can be called.

---


### resetCount

```
public void resetCount()
```

:   Causes this progress monitor to reset it's internal counter to 0.

    :   **Specified by:**: `resetCount` in interface `MutableProgressMonitor` **Overrides:**: `resetCount` in class `AbstractProgressMonitor`
    :   **See Also:**: `increment()`, `AbstractProgressMonitor.getCount()`

---


### increment

```
public void increment()
```

:   Causes this progress monitor to increment an internal counter.

    :   **Specified by:**: `increment` in interface `MutableProgressMonitor` **Overrides:**: `increment` in class `AbstractProgressMonitor`
    :   **Throws:**: `java.lang.IllegalStateException` - if this call causes the internal counter to exceed the provided total. **See Also:**: `AbstractProgressMonitor.getCount()`

---


### getProgress

```
public java.lang.Double getProgress()
```

:   Returns a double precision number indicating the completed percentage
    of the process being monitored.

    :   **Returns:**: a double precision number indicating the completed percentage of the process being monitored.

---


### complete

```
public void complete()
```

:   Sets the internal counter equal to the provided total. Subsequent calls
    to `getProgress()` will return 100.

---


### asUnmodifiable

```
public ProgressMonitor asUnmodifiable()
```

:   Returns this progress monitor as an immutable object. This allows
    the contents of this progress monitor to be queried without the
    possibility of modification.

    :   **Returns:**: this progress monitor as an immutable object.


---


|  |  |  |
| --- | --- | --- |
| **Overview** \| **Package** \| **Class** \| **Use** \| **Tree** \| **Deprecated** \| **Index** \| **Help** | |  |
| **PREV CLASS**   **NEXT CLASS** | **FRAMES**    **NO FRAMES**     **All Classes** |  |
| SUMMARY: NESTED \| FIELD \| CONSTR \| METHOD | DETAIL: FIELD \| CONSTR \| METHOD |  |


---
